# Gene essentiality profiling reveals a novel determinant of stresses preventing protein aggregation in *Salmonella*

**DOI:** 10.1080/22221751.2022.2081618

**Published:** 2022-06-04

**Authors:** Zuoqiang Wang, Siqi Zhu, Congcong Li, Lin Lyu, Jingchen Yu, Danni Wang, Zhihong Xu, Jinjing Ni, Beile Gao, Jie Lu, Yu-Feng Yao

**Affiliations:** a Laboratory of Bacterial Pathogenesis, Department of Microbiology and Immunology, Shanghai Jiao Tong University School of Medicine, Shanghai, People’s Republic of China; b CAS Key Laboratory of Tropical Marine Bio Resources and Ecology, Guangdong Key Laboratory of Marine Materia Medica, South China Sea Institute of Oceanology, Chinese Academy of Sciences, Guangzhou, People’s Republic of China; c Southern Marine Science and Engineering Guangdong Laboratory, Guangzhou, People’s Republic of China; d University of Chinese Academy of Sciences, Beijing, People’s Republic of China; e Department of Infectious Diseases, Shanghai Ruijin Hospital, Shanghai, People’s Republic of China; f Shanghai Key Laboratory of Emergency Prevention, Diagnosis and Treatment of Respiratory Infectious Diseases, Shanghai, People’s Republic of China

**Keywords:** *Salmonella* Typhimurium, Tn-Seq, stress adaptation, fitness determinants, protein aggregation

## Abstract

Adaptation to various stresses during infection is important for *Salmonella* Typhimurium virulence, while the fitness determinants under infection-relevant stress conditions remain unknown. Here, we simulated conditions *Salmonella* encountered within the host or in the environment by 15 individual stresses as well as two model cell lines (epithelium and macrophage) to decipher the genes and pathways required for fitness. By high-resolution Tn-seq analysis, a total of 1242 genes were identified as essential for fitness under at least one stress condition. The comparative analysis of fitness determinants in 17 stress conditions indicated the essentiality of genes varied in different mimicking host niches. A total of 12 genes were identified as fitness determinants in all stress conditions, including *recB*, *recC*, and *xseA* (encode three exonuclease subunits necessary for DNA recombination repair) and a novel essential fitness gene *yheM*. YheM is a putative sulfurtransferase subunit that is responsible for tRNA modification, and our results showed that *Salmonella* lacking *yheM* accumulated more aggregates of endogenous protein than wild-type. Moreover, we established a scoring scheme for sRNA essentiality analysis and found STnc2080 of unknown function was essential for resistance to LL-37. In summary, we systematically dissected *Salmonella* gene essentiality profiling and demonstrated the general and specific adaptive requirements in infection-relevant niches. Our data not only provide valuable insights on how *Salmonella* responds to environmental stresses during infections but also highlight the potential clinical application of fitness determinants in vaccine development.

## Introduction

*Salmonella* species are facultative intracellular pathogens that cause localized or systemic infections resulting in high morbidity and mortality worldwide [1]. *Salmonella enterica* serovar Typhimurium (*S.* Typhimurium) is a key serotype that poses a great threat to human health. This pathogen can cause diarrhoea and systemic diseases in both animals and humans by eliciting inflammatory changes in the intestinal epithelium and proliferating in macrophages [2,3].

As a major food-borne pathogen, *S*. Typhimurium encounters multiple stresses. In order to cause infection in the host, *S*. Typhimurium must first survive under several stresses during food processing. These stresses include physical treatments such as heat or cold; and intrinsic factors in food such as desiccation [4]. Upon entering the host through ingestion of contaminated food, *S.* Typhimurium overcomes many levels of mammalian host defences to successfully colonize and survive inside the host. Prior to invasion into host cells, *S.* Typhimurium survives an extreme acidic environment within the stomach, which can be considered as one of the host’s first barrier [5]. Entry of *S.* Typhimurium takes place after reaching the epithelial cell in the intestine, followed by inflammation due to infiltration of the immune cells such as macrophages [6]. Once in the intestine, *S.* Typhimurium encounters intestinal bile salts, limited iron (Fe^2+^), low oxygen tension conditions and increased osmolarity [7]. The macrophages also present many hazards to the *S.* Typhimurium, including nutrient limitations, as well as exposure to peroxidative stress and various host antimicrobial peptides [7].

*S*. Typhimurium carries various genes that permit survival under different environmental stress conditions, as well as during the invasion of epithelial cells, colonization of the gastrointestinal tract, and replication within macrophages. Therefore, the identification and comparison of genetic requirements for survival under different niches will improve our understanding of *S*. Typhimurium pathogenesis.

Transposon-insertion sequencing (Tn-seq) combines large-scale transposon mutagenesis with high-throughput sequencing to determine bacterial genetic requirements on a genome-wide scale [8,9]. Over the last decade, several Tn-seq-based strategies have been used to analyze genetic requirements and fitness in *S.* Typhimurium individually both *in vivo* and *in vitro* [10–15]. However, because of different strain backgrounds, varying transposon library quality and/or different analytical approaches, it is difficult to assess the congruence of gene essentiality across different stresses. Therefore, the integrated *S.* Typhimurium gene essentiality profiling under different niches remains to be established.

To comprehensively investigate the stress adaptive mechanisms of the *S.* Typhimurium during infection, we created a saturated transposon mutant library to determine the essentiality of each gene under different conditions. We chose 12 conditions to mimic stresses *S.* Typhimurium encountered during different infection stages and three stresses in food processing to reflect the integrity of the gene essentiality profiling. In addition, two model cell lines were utilized to mimic two major infection processes, i.e. the invasion of intestinal epithelial cells and proliferation in macrophages. We profiled conditionally essential genes and small non-coding RNAs (sRNAs) under all 17 conditions. Our findings provide an integrated resource to understand the highly relevant gene essentiality profiling involved in the survival of *S*. Typhimurium in different physiological niches.

## Materials and methods

### Bacterial strains and culture conditions

*Salmonella* Typhimurium strain 14028s was used to create the transposon insertion library. *Escherichia coli* strain β-2163 was grown at 37°C in Lysogeny Broth (LB) medium supplemented with 57 μg/ml 2,6-diaminopimelic acid. The LB medium was used as a rich medium, whereas nutrient agar plates contained 1.5% (w/v) agar supplemented with antibiotics as required. The antibiotics used were 100 μg/ml ampicillin and 17 μg/ml chloramphenicol. All the strains were stored at −80°C in LB medium containing 15% glycerol.

### Construction of the transposon mutant library

The *S*. Typhimurium transposon mutant library used in this study was constructed using a derivative of the *Himar1* Mariner transposon [8] delivered by pCat_Mariner. *Escherichia coli* strain β-2163 was used as the donor strain to transfer the suicide vector into *S*. Typhimurium strain 14028s by conjugation. Briefly, *E. coli* strain β2163 harbouring pCat_Mariner was grown overnight in LB supplemented with 100 μg/ml ampicillin and 57 μg/ml 2,6-diaminopimelic acid. The recipient *S*. Typhimurium strain was grown in LB at 37°C overnight. Equal volumes (1 ml) of donor and recipient were mixed, centrifuged, washed in 10 mM MgSO_4_, and re-suspended in 1 ml PBS. Then, 200 μl of the mating mixture was dropped onto a single sterile nitrocellulose filter on an LB agar plate. After 5 h of conjugation at 37°C, the cells on the filter were washed in 2 ml PBS and plated on LB agar containing 17 μg/ml chloramphenicol. After 14 h at 37°C, approximately 200,000 mutants were collected onto LB medium containing 15% glycerol. The library was stored in aliquots at −80°C.

### *In vitro* selection of the transposon mutant library

Before the screening, an aliquot of the transposon libraries was taken from stock in −80°C, thawed at room temperature, and diluted 1:10 in LB broth. The library was recovered at 37°C with shaking at 250 rpm for 1 h. Recovered library contained 5 × 10^9^ CFUs of mutants per ml. Stress conditions in LB broth were performed on recovered library as follows: Hydrogen peroxide stress (H_2_O_2_), 5 × 10^8^ mutants were subjected to LB broth with 2.5 mM H_2_O_2_ and grew for 2 h at 37°C; bile salt stress (Bile), 5 × 10^8^ mutants were subjected to LB broth with 10% (w/v) ox bile (sodium cholate hydrate) and grew for 2 h at 37°C; osmotic stress (osmolarity), 5 × 10^8^ mutants were subjected to LB broth with 4% (w/v) NaCl and grew for 4 h at 37°C; iron limitation stress (Low Fe^2+^), 5 × 10^6^ mutants were subjected to LB broth with 0.2 mM 2,2’-dipyridyl and grew for 6 h at 37°C [16]; cold stress (Cold), 5 × 10^8^ mutants were transferred to air bath incubator maintained at 4°C for two days without shaking; heat stress (heat), 5 × 10^6^ mutants were transferred to air bath incubator and grew at 45°C for 6 h; anaerobic stress (anaerobiosis), 5 × 10^8^ mutants were transferred into 50 ml centrifuge tube containing 49.5 ml of LB broth, screwed tight and incubated in 37°C overnight without agitation. For nitric oxide (NO) stress, the recovered library was washed twice in phosphate-buffered saline (PBS), and then 5 × 10^8^ mutants were incubated in PBS with addition of NO donor Spermine NONOate to a final concentration of 250 μM at 37°C for 6 h [17]. For LL-37 stress, the recovered library was washed twice in M9CA broth (2.0 g/l casamino acid, 6.8 g/l Na_2_HPO_4_, 3.0 g/l KH_2_PO_4_, 1.0 g/l NH_4_Cl, 0.5 g/l NaCl), and then 5 × 10^6^ mutants were incubated in M9CA broth with 40 μg/ml LL-37 at 37°C for 2 h. For acidic pH, the recovered library was washed twice in EG medium (2.0 g/l Citric acid, 10.0 g/l K_2_HPO_4_, 2.3 g/l NaNH_4_HPO_4_, 0.1 g/l MgSO_4_, 0.4% w/v glucose)at pH 7.7, 5 × 10^6^ mutants were cultured in pH 7.7 EG medium for 3 h, and then challenged in EG medium at pH 3.3 for 3 h at 37°C [18]. For growth in a medium with limited phosphate (P), carbon (C), nitrogen (N), and PCN, modified minimal MOPS (3-(N-Morpholino) propane-sulfonic acid)-salts medium (MS medium) was used and supplied with variant amounts of P, C, and N. The composition of MS medium used in this study was as follows: 40 mM MOPS (pH 7.4, adjusted with KOH); 4 mM Tricine (pH 7.4, adjusted with KOH); 50 mM NaCl; 10 μM FeCl_3_; 0.276 mM K_2_SO_4_; 0.5 μM CaCl_2_ and 0.5 mM MgCl_2_. Non-limiting amounts of P, C, and N were added at the following final concentrations: 15 mM KH_2_PO_4_, 0.4% w/v glucose, and 15 mM NH_4_Cl, respectively. Limiting amounts of P, C, and N used in starvation-induction assays were added at the following final concentrations: 0.1125 mM KH_2_PO_4_, 0.04% w/v glucose, and 0.6 mM NH_4_Cl, respectively [19]. 5 × 10^8^ mutants were washed twice in MS medium and grew in variants of starving conditions at 37°C for four days. For desiccation stress, recovered library was diluted to 5 × 10^8^ CFUs per ml and 400 μl were placed at the centre of petri plates (90 × 15 mm size) and air-dried with the lid open inside a biosafety hood with the blower on, the bacteria were scraped after 4 h.

A total of three independent biological replicates, each with approximately 70,000 individual insertion mutants, were used to ensure the robust coverage of the library.

### Selection in mouse macrophage-like RAW 264.7 cells and HeLa cells

The RAW 264.7 cells were maintained in Dulbecco’s modified Eagle’s medium supplemented with 10% (v/v) fetal bovine serum, and were used for infections at passage 10 or less. Three serial infections were performed in the macrophages. First, two rounds were conducted at a multiplicity of infection (MOI) of 10, whereas the third round was carried out at an MOI of 1. Cultures of the transposon library grown overnight in LB containing 17 μg/ml chloramphenicol were opsonized in 15% normal mouse serum and PBS at 37°C for 15 min. The bacteria were pelleted and resuspended in PBS and used to infect 5 × 10^6^ macrophage cells at an MOI of 10 or 5 × 10^7^ cells at an MOI of 1 for 60 min. Cells were rinsed in PBS, treated with Dulbecco’s modified Eagle’s medium supplemented with 10% (v/v) fetal bovine serum and 100 μg/ml gentamicin for 2 h and then maintained in 25 μg/ml gentamicin for the duration of the infection. After 24 h, macrophages were lysed with 1% Triton X-100, and then, the bacteria were harvested by centrifugation and inoculated into 5 ml LB containing 17 μg/ml chloramphenicol for 12 h of growth at 37°C. The output from each round was used for the next round of infection following opsonization. Bacterial cells from each round were harvested for genomic DNA extraction.

The same experiment was performed in HeLa cells with some modifications, including the MOI for the third round being 10, a lack of opsonization in the assay and the cells were infected for 2 h.

### Tn-seq DNA sample preparation and data analysis

The Tn-seq DNA sample preparation and amplification were carried out as previously described [8,20]. Genomic DNA was extracted from each of the mutant pools and digested with the *Mme* I restriction enzyme (New England Biolabs). After being separated by agarose gel electrophoresis, transposon-sized fragments were extracted from the gel and ligated to a double-stranded DNA adaptor by overnight incubation at room temperature. The ligated samples were subsequently used as templates and PCR amplified for 22 cycles using a transposon-specific and an adaptor-specific primer. The resulting 121-bp products, containing barcodes to identify the individual mutant pools, were purified and sequenced on an Illumina system. The deep sequencing data were analyzed using the INSeq_pipeline3 [20]. First, the raw reads were sorted to each sample using the sample-specific barcodes. Then, the sequences were analyzed to identify transposon inverted repeats and trimmed to remove the transposon sequence. The remaining 14- to 16-bp transposon-junction sequences were then analyzed. The junction sequences were mapped to the *S*. Typhimurium strain 14028s’ chromosomes and plasmid using Bowtie-0.12.7. The read numbers of each insertion site were counted and assigned to the coding or intergenic region. In addition, insertions with total read numbers of less than, or equal to, three were further filtered. To compare the changes in the relative abundance of each mutant gene between different samples, the total read number of each sample was normalized to counts per million reads. After normalization, the log-ratio of output read number to input read number for each gene was calculated to assess the fitness contribution of the specific gene. A Z-test was performed to identify the fitness genes with significantly different log-ratios among the three replicates [8].

### Generation of knock-out strains

All the mutants derived from *S*. Typhimurium strain 14028s were constructed using the λ Red recombinase system. Briefly, the *S*. Typhimurium strain was transformed with pKD46, which contains genes coding for the arabinose-induced λ Red recombinase system that promotes recombination between linear pieces of DNA (PCR products) and the host chromosome. The recombination is based on short stretches of homology (50 nucleotides) between the linear DNA and the site of recombination. The PCR products used for knocking out the target genes were amplified, gel extracted, and electroporated into competent strain 14028s containing pKD46 prepared in the presence of arabinose. The deletion mutants were screened using antibiotics. They were verified by PCR using primers adjacent to the genic region, followed by sequencing.

### Aggregated proteins isolation and SDS-PAGE separation

Aggregated proteins were isolated as previously described [21] with minor modifications. Aliquots of bacterial cultures with the same optical density (16 ml of culture of OD_600_ = 1.0) were incubated in a water bath at different temperatures (37°C, 42°C and 48°C) for 30 min. After heat shock treatment, cultures were then rapidly cooled on ice and centrifuged at 5000 g and 4°C for 10 min. Pellets were resuspended in 40 μl buffer A (10 mM KH_2_PO_4_, pH 6.5, 1 mM EDTA, 20% w/v sucrose, 1 mg/ml lysozyme) and incubated for 30 min on ice. Cell lysate was added with 360 μl buffer B (10 mM KH_2_PO_4_, pH 6.5, 1 mM EDTA) followed by sonication. Intact cells and cell debris were removed by centrifugation at 2000 g and 4°C for 15 min. Membrane and aggregated proteins were isolated by subsequently centrifugation at 15,000 g for 20 min. Supernatants were collected to indicate protein concentrations. The pellet fractions were resuspended with 400 μl buffer B by brief sonification and centrifugation 15,000 g, 20 min, 4°C. After removing supernatants, aggregated proteins were washed twice with 400 μl buffer C (buffer B with 2% NP40) to dissolve membrane proteins. NP40-insoluble pellets were washed with 400 μl buffer B and resuspended in 50 μl buffer B. Gel electrophoresis of the aggregated protein was carried out using 12% SDS-PAGE and stained with Coomassie brilliant blue.

## Results

### Construction of *S.* Typhimurium transposon mutant library and identification of fitness determinants

**Figure 1.**
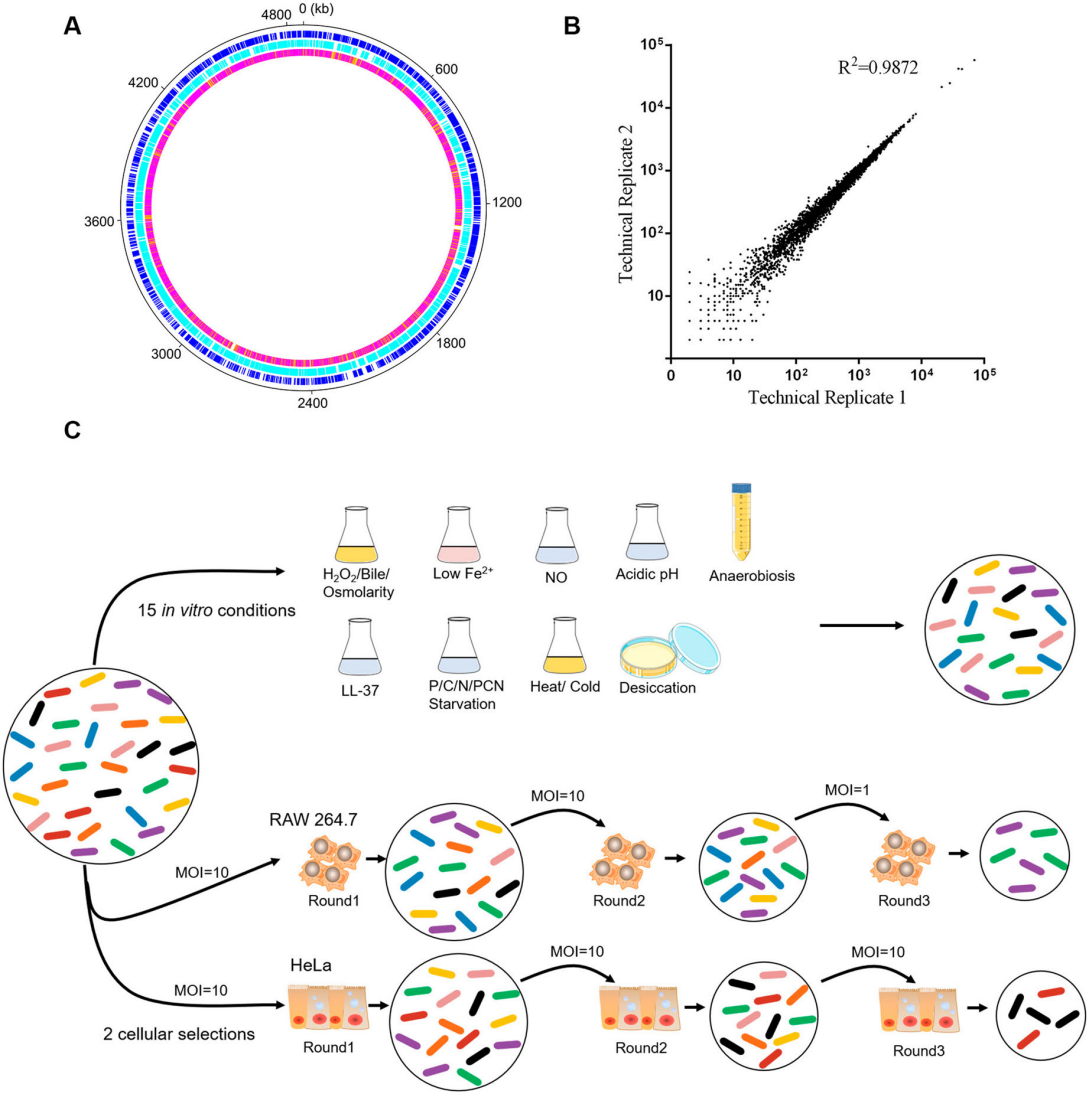
**Overview and stress-based selection of the *S*. Typhimurium transposon mutant library.** (a) Circular diagram showing the distribution of transposon mutants within the *S*. Typhimurium genome in the library. The outer track (black) showed the full *S*. Typhimurium genome, the next two tracks (blue and cyan) showed the distributions of genes by DNA strand, and the inner track showed the number of reads per kb of the genome (white, 0 reads; orange, ≤ 10; magenta, ≤ 100; red, > 100). (b) Reproducibility of experimental protocols. The normalized reads of a single gene from two technical replicates were plotted. (c) Schematic representation of the Tn-Seq carried out *in vitro* and in model cell lines used in this study. In total, 10^6^–10^8^ colony forming units (CFUs) of the *S*. Typhimurium transposon mutant library were selected under 15 *in vitro* conditions. The RAW 264.7 and HeLa cells were infected by 5 × 10^7^ CFUs of the mutant library at the indicated MOIs for three rounds. The bacterial cells were collected and amplified after selection.

*S*. Typhimurium is an adaptable and robust pathogen that thrives under several hostile conditions. To identify fitness determinants under different conditions, we generated a random transposon insertion mutant library in *S*. Typhimurium using a mariner transposon which is restricted to insertions at TA dinucleotides. The relative abundance of insertion sites in the transposon mutant library was examined using high-throughput sequencing. The original mutant pool contained ∼70,000 independent insertions at TA sites (30.0% saturation), of which ∼60,000 were in coding sequences (CDS) and ∼10,000 in intergenic regions (Dataset S1). The insertion sites, with an average density of ∼15 insertions/kb, revealed no apparent bias among chromosomes (Figure 1(a)). Comparisons of the pools’ compositions across technical replicates indicated a high degree of reproducibility (Figure 1(b)).

Here, we subjected the library to three conditions relevant to food processing, including desiccation, cold and heat. Additionally, we exposed the library to 12 stressful conditions to mimic *in vivo* environment when *S*. Typhimurium colonize and proliferate in different niches. These conditions are acidic pH that *S*. Typhimurium must resist in stomach; anaerobiosis, bile, high osmolarity and low iron (Fe^2+^) in intestine; hydrogen peroxide (H_2_O_2_), nitric oxide (NO), cationic antimicrobial peptides LL-37 (the only member of cathelicidin family of peptides expressed in humans, which consists of 37 amino acid residues with the two leading residues being leucines); as well as phosphorus (P), nitrogen (N), carbon (C) starvation and simultaneous starvation of P, C, and N (PCN) in macrophages (Figure 1(c)). In addition, we employed HeLa cells and mouse macrophage RAW 264.7 cells (Figure 1(c)) to determine fitness determinants during *Salmonella* invasion of intestinal epithelial cells and proliferation in macrophages.

After the library was selected under each condition, genomic DNA was prepared from the output samples for high-throughput sequencing. The distribution and abundance of transposon insertion sites in the output pools were determined. Statistics from the sequencing analysis of the output pools are summarized in Dataset S1.

The ratio of output to input relative abundance (fold change, FC) for each gene were calculated. Only genes with average log base 2 FC transformations (log_2_ FC) of read counts less than −1.0 and *p* values less than 0.05 were considered “fitness determinants” under the *in vitro* conditions. For cellular selections, because the transposon library was serially passaged in cells for multiple rounds and the selection pressure increased during each round, the weighted average log_2_ FC of each gene (the weight of each round was 6:3:1) was calculated instead of the normal average to avoid the bias caused by some transposon mutants’ enormous proportion in round 3. Genes with weighted average log_2_ FC of three replicates less than −1.0 and *p* values less than 0.05 were designated as “fitness determinants.” The number of reads, transposon insertion numbers, average log_2_ FC (weighted) and *p* values per gene are summarized in Dataset S2.

### Genome-wide analysis of fitness determinants for stress adaptation

**Figure 2.**
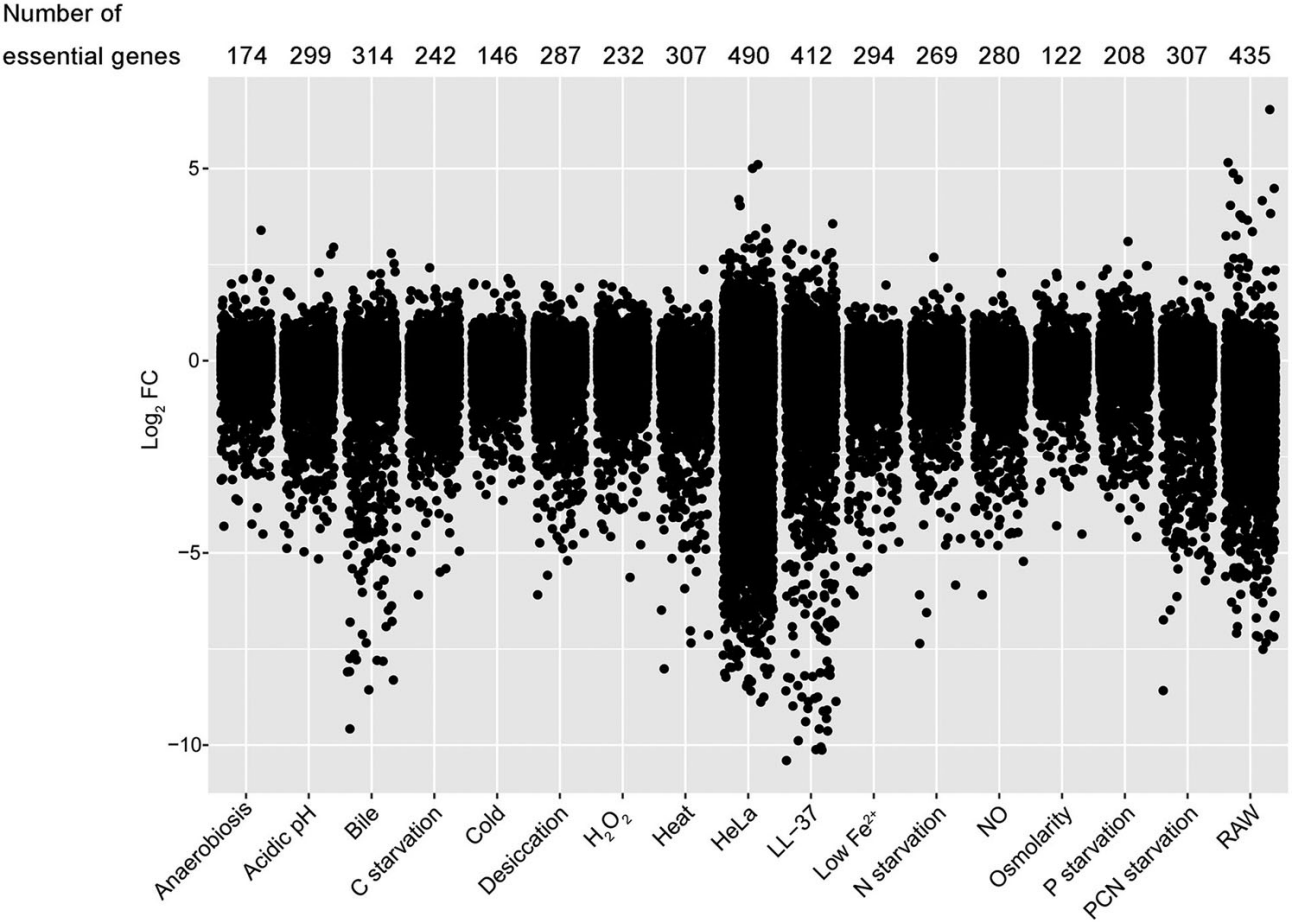
**Visualization and comparison of gene essentiality under 17 stress conditions.** Jitter plot showing the extent of mutant fitness costs calculated using the mean log_2_ FC across three replicates for each condition. The number of fitness determinants under each condition was labelled above the plot.

To compare the differences in the extent of requirement for each gene under 17 conditions, fitness contribution per gene was analyzed by calculating the average log_2_ FC in transposon abundance from three replicates. The smaller the value of the log_2_ FC per gene, the greater the fitness contribution. *S.* Typhimurium required more genes with high essentiality in two cell lines and two *in vitro* stresses (LL-37 and bile treatment) tested, whereas, under mild stress conditions, such as anaerobiosis, cold, high osmolarity, and P starvation, both the number of fitness determinants and the absolute value of FC values are less (Figure 2). A certain gene may be essential under several conditions but have various effects on fitness. For example, under osmotic stress conditions, genes encoding acridine efflux pumps, namely *acrA* and *acrB*, had fitness values of −1.48 and −1.66, respectively. However, under bile salt stress condition, these genes had fitness values of −7.78 and −6.80, respectively, reflecting more than 32-fold greater effects (Dataset S2). Overall, mutant fitness costs were greater for direct antimicrobial stresses, such as LL-37, bile, NO, and H_2_O_2_, than for bacteriostatic ones, like anaerobiosis, cold and high osmolarity.

To systematically analyze the differences and similarities among the gene dependency under 17 conditions, the output libraries were hierarchically clustered using their log_2_ FC values per gene to compare correlations on a genome-wide scale. The samples from treatment with four kinds of nutrient starvation (P, C, N, and PCN) were the most correlated (Figure S1). Similarly, other samples from treatment with *in vitro* stresses, such as NO, desiccation, low Fe^2+^, and anaerobiosis, displayed high levels of overlap in fitness determinants (Figure S1). By contrast, some stresses that *S.* Typhimurium encountered in the same niche had only low levels of overlap in fitness determinants (such as H_2_O_2_ and LL-37 treatments), indicating that *S.* Typhimurium utilizes different mechanisms to respond to these stresses.

**Figure 3.**
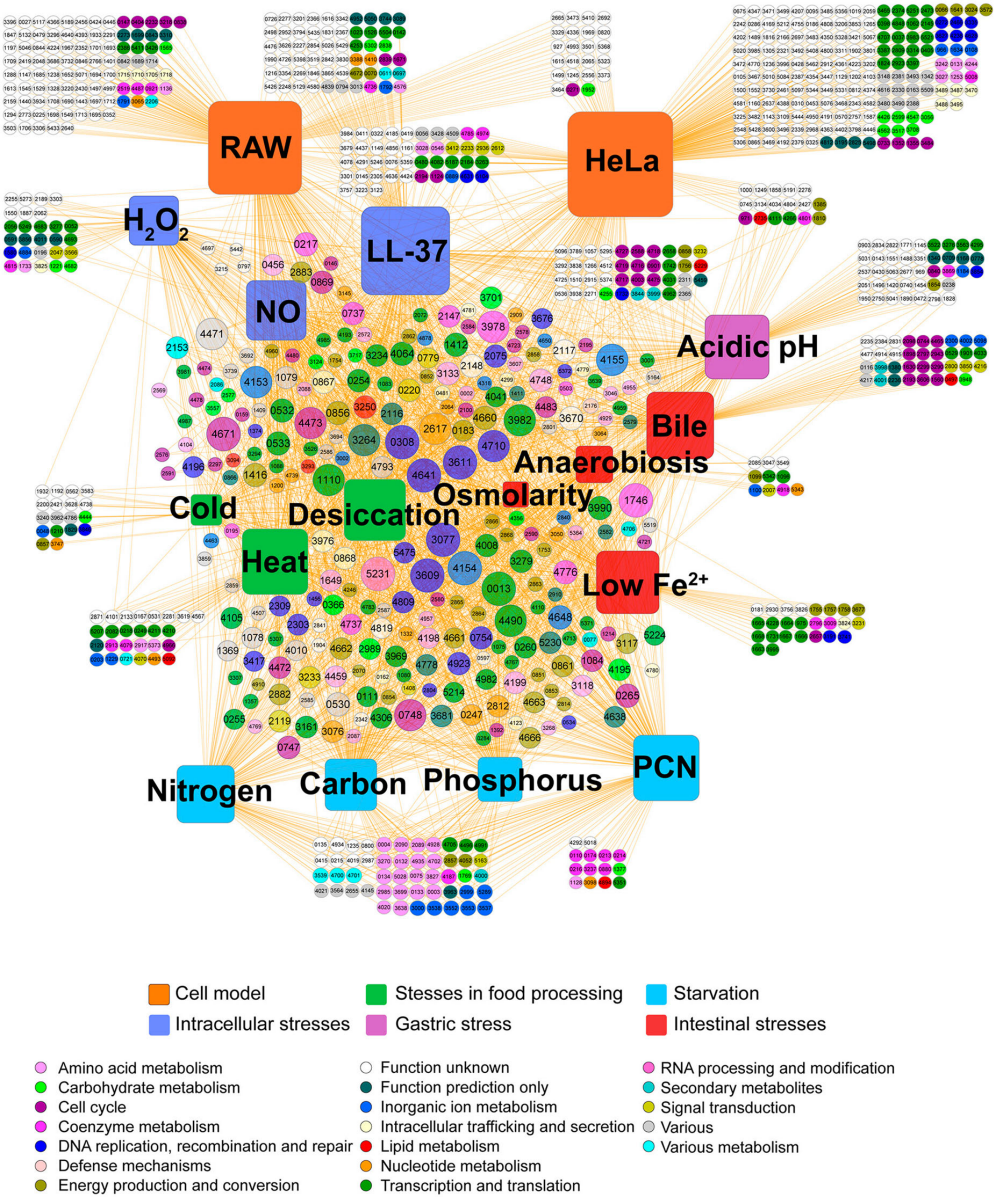
**Interaction network between fitness determinants and conditions.** A total of 1242 fitness determinants were visualized in a gene-condition interaction network. Conditions were represented as rounded squares and genes are represented as circles. The numbers in the circles correspond to the STM14 genome number. The sizes of the squares corresponded to the number of fitness determinants under each stress condition. The size of the circles increased with the number of interactions. Genes were colour-coded according to their functional category.

We then determined the genes that were essential under each condition and visualized them in an interaction network (Figure 3). In total, 1242 genes were identified as essential for fitness under at least one condition. The central part of the network was characterized by genes that were essential for multiple conditions, and these genes were related to a variety of biological functions, including DNA recombination and repair, amino acid metabolism, transcription, and translation, etc. Most of these genes were shared by several conditions, suggesting that *Salmonella* required gene networks to respond to different stresses. The fitness determinants unique in each condition were located away from the centre of the network. Approximately 40% of genes were essential for a specific condition. Consistent with Figure 2, greater numbers of fitness determinants were selected after serial infections of the two cell lines, showing that *S.* Typhimurium required more genes for invasion of HeLa and proliferation in RAW 264.7 cells. Since phagosomes or phagolysosomes present an integrated stress to intracellular bacteria, it is understandable that *Salmonella* requires more genes to handle these compounded intracellular hazards in macrophages than other individual stresses. Another critical factor affecting the virulence of *S.* Typhimurium is the ability to invade epithelial cells. This condition required not only the greatest number of fitness determinants (*n* = 490), but also 59.0% (289/490) of these mutants had fitness values less than −5 (Figure 2), indicating that these genes were extremely indispensable for epithelial invasion by *Salmonella*.

### Comparison of fitness determinants among nutrient starvation conditions

*Salmonella* requires both unique and overlapping sets of genes to resist starvation of P, C, N, or PCN [19]. Our results revealed that the four nutrient starvation conditions had the closest relevance of the fitness gene requirement among all the 17 conditions (Figure S1). Interestingly, PCN starvation had some specific genetic requirements compared with the other three individual starvation conditions (Figure 3). Therefore, we hypothesized that even though the fitness determinants of the four starvation conditions showed a high correlation, the fitness cost of the compounded starvation was much more severe than those of any single-source starvation conditions.

**Figure 4.**
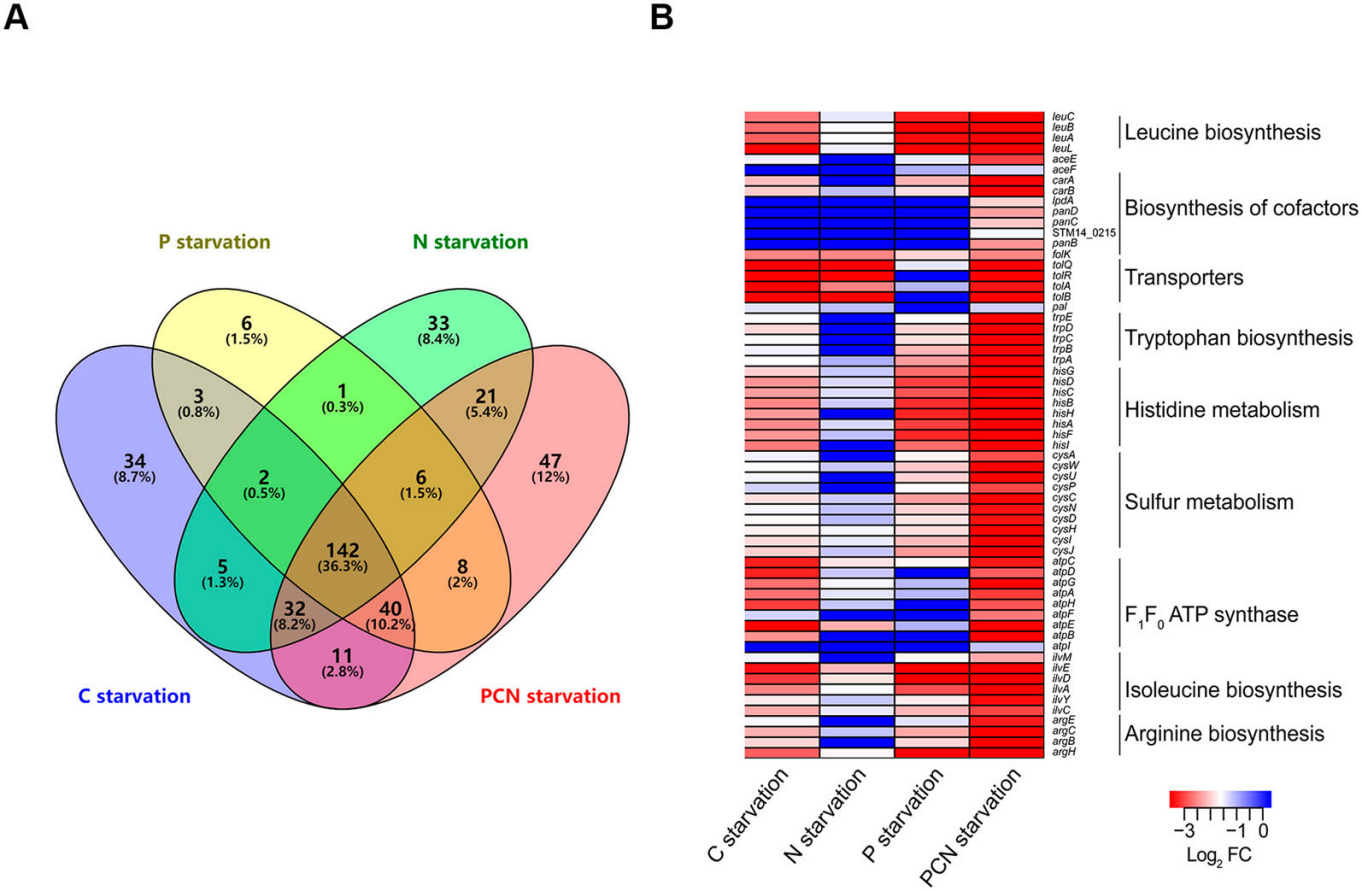
**Analysis of fitness determinants under starvation stresses.** (a) The Venn diagrams showed the numbers of fitness determinants under nutrient stress conditions. (b) Heatmap highlighting biologically relevant clusters. A cluster analysis was performed using the log_2_ FC values of the 61 genes that were shown to be essential under at least one starvation condition.

To test this hypothesis, we compared the fitness genes under the four nutrient starvation conditions. A total of 391 genes were essential under at least one starvation condition, and 142 (36.3%) genes overlapped under all four starvation conditions (Figure 4(a)), indicating that these genes were generally required to respond to nutrient limitations in *Salmonella*. Moreover, 307 (78.5%) genes were identified during PCN starvation (Figure 4(a)), indicating that *S.* Typhimurium required more genes to overcome the compounded starvation conditions than individual starvation conditions. Furthermore, a heatmap was constructed to represent levels of gene essentiality using log_2_ FC, and fitness determinants were compared using clustered pathways (Figure 4(b)). The levels (absolute value of Log_2_ FC) of gene essentiality in arginine, isoleucine, leucine, and tryptophan biosynthesis, as well as in pyruvate, histidine, and sulphur metabolism, under PCN starvation conditions were greater than that under single nutrient starvation conditions (Figure 4(b)). Therefore, *S.* Typhimurium required more genes with higher essentiality to overcome the compounded nutrient deficiency during PCN starvation.

### General and specific adaptive requirements in mimicked niches during *S.* Typhimurium infection

*S.* Typhimurium encountered multiple stresses when resided in three *in vivo* niches, including stomach, intestine, and macrophages. Based on the actual environmental stresses in these niches [7], we defined acidic pH as gastric stress; bile, low Fe^2+^, anaerobiosis, and high osmolarity as intestinal stresses; H_2_O_2_, NO, LL-37, and PCN starvation as intracellular stresses. To explore the specific and general genetic requirements in these niches, we get the collection of fitness determinants from gastric stress, intestinal stresses, and intracellular stresses, and then clustered them by KEGG enrichment analysis. Genes involved in ATP biosynthesis, ATP-binding cassette-type transporter (ABC transporter), and amino acid biosynthesis were enriched.

**Figure 5.**
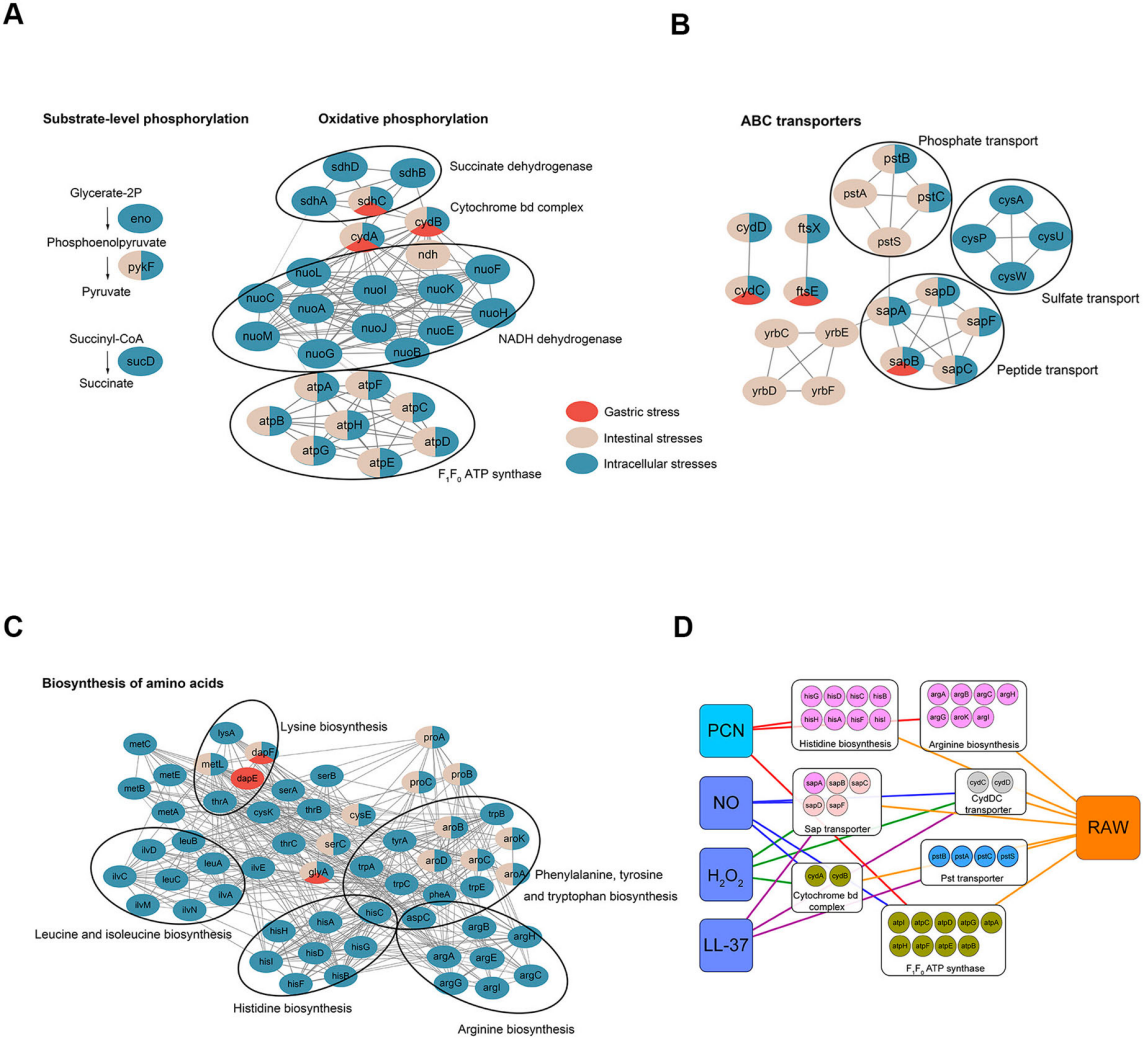
**Differential essentiality analysis of genes between stomach-, intestine- and intramacrophage-mimicking niches.** Analysis of essential gene networks identified in stomach-, intestine- and intramacrophage-mimicking niches. The pathways and protein complex included ATP synthesis pathway (a); ABC transporter (b); amino acids synthesis pathway (c). Genes were represented as ellipses and colour-coded according to their essentiality in mimicked niches. Gene interactions were indicated by the grey line. (d) A pathway-specific subnetwork indicating the shared fitness determinants between four intramacrophage-mimicking conditions (H_2_O_2_, NO, LL-37, and PCN starvation) and macrophage RAW 264.7 cells. Genes were colour-coded the same as in Figure 3.

There are two aerobic ATP biosynthetic pathways in prokaryotic cells, oxidative phosphorylation, and substrate-level phosphorylation. Oxidative phosphorylation is the main pathway for ATP synthesis, while substrate-level phosphorylation is a supplementary pathway. In general, bacterial oxidative phosphorylation is composed of electron-transfer complexes and F_1_F_0_ ATP synthase. The electron-transfer complexes are membrane-embedded respiratory enzymes, which first generate an electrochemical gradient of a proton across the membrane, then F_1_F_0_ ATP synthase uses the proton gradient to provide the necessary energy to drive the ATP synthesis [22]. Small amount of ATP molecules is synthesized in glycolytic pathway, which is called substrate-level phosphorylation. By analyzing the essentiality of genes coding three electron-transfer complexes (NADH dehydrogenase, succinate dehydrogenase, and cytochrome *bd* complex) and F_1_F_0_ ATP synthase in oxidative phosphorylation, as well as three key glycolytic enzymes (Eno, PykF, and SucD) involved in substrate-level phosphorylation, we found neither F_1_F_0_ ATP synthase nor enzymes in substrate-level phosphorylation were essential in gastric stress (Figure 5(a)). In intestinal stresses, F_1_F_0_ ATP synthase was essential, while certain electron-transfer complexes were dispensable (Figure 5(a)). In intracellular stresses, all the components in oxidative phosphorylation and substrate-level phosphorylation were essential (Figure 5(a)). These results present the diverse levels of ATP demand in response to stresses in different niches.

Then we analyzed the roles of ABC transporters in different niches and found that *S.* Typhimurium required certain transporter for a specific niche. For example, Yrb transporter was essential in intestinal conditions and Cys transporter was required in intracellular conditions (Figure 5(b)). Some transporters have a general requirement in multiple niches, such as Sap transporter, Pst transporter, and CydDC (Figure 5(b)).

The Sap transporter, consisting of five proteins, is involved in antimicrobial peptide resistance [23], which is consistent with our Tn-seq data in which the *sap* locus was required for *S.* Typhimurium after exposure to LL-37 (Dataset S2). The Sap transporter was also found to be essential under low Fe^2+^ stress condition, which had not been reported previously. In nontypeable *Haemophilus influenzae*, haem utilization is dependent on Sap transporter that binds haem to SapA [24]. Since haem can be a main iron source for pathogenic bacteria, we propose that as a peptide transport protein, the Sap transporter could not transport iron directly but could regulate iron uptake by transporting Fe-containing compounds, like haem or hemoproteins.

In *Escherichia coli*, *cydC*, and *cydD* encode a heterodimeric ABC transporter that is required for the synthesis of cytochrome *bd* complex [25]. As a result, *cydC* and *cydD* shares similar niche-based requirements with *cydA* and *cydB* (Figures 5(a and b)). CydDC also mediates glutathione (GSH) and cysteine transport across the *E. coli* cytoplasmic membrane [26], and GSH is a major biological antioxidant that helps maintain redox balance in prokaryotes cells. Because CydDC protects *E. coli* against NO [27], it seems that the ability of CydDC to transport GSH provides an auxiliary protection besides that of cytochrome *bd* complex. Consequently, the fitness effects of *cydC* and *cydD* (log_2_ FC = −4.2 and −3.53, respectively) under NO stress condition were much greater than those of *cydA* and *cydB* (log_2_ FC = −2.45 and −0.86, respectively; Dataset S2).

Methionine is essential for many important cellular and biosynthetic functions, and methionine biosynthesis is required for *S.* Typhimurium virulence in the host [28]. By analyzing the essentiality of biosynthesis of different amino acids in distinct niches, we found not only methionine but also several other amino acids biosynthesis was essential, like histidine and arginine biosynthesis in an intracellular niche (Figure 5(c)).

Considering the collection of fitness determinants in simulated niche-specific stresses may not truly reflect the genetic requirements in *in vivo* niche, we compared fitness determinants in four intracellular niche-mimicked *in vitro* stresses with those in mouse macrophage-like cell line RAW 264.7 by a pathway-specific subnetwork (Figure 5(d)). The pathways and components mentioned above, including histidine and arginine biosynthesis, Sap transporter, CydDC transporter, cytochrome *bd* complex and F_1_F_0_ ATP synthase, were all identified as essential in macrophage-like cell line RAW 264.7, which supports the reliability of our analytic strategy. Moreover, by this means, we could elaborate the mechanism why certain genes are essential under a specific niche. Taken the genes encoding histidine and arginine biosynthesis as an example, we can conclude that these two pathways were essential for *Salmonella* survival in macrophages because of the nutrient limitations in this niche. Furthermore, we confirmed the decreased fitness of the *atpB*, *sapB*, *argC* and *argH* deletion mutants in RAW 264.7 cells (Figure S2).

An exceptional case is *Salmonella* pathogenicity island (*SPI*)-*2*, which is required for *Salmonella* survival inside macrophages [29]. Our data showed that 25 of the 33 *SPI-2* genes were essential for intramacrophage survival, and 20 *SPI-2* genes were exclusively essential for intramacrophage survival rather than under *in vivo* mimicking conditions, including H_2_O_2_, LL-37 NO, and PCN starvation (Figure S3A). Interestingly, the *SPI-2* genes were not induced at the transcriptional level under single infection-relevant situations, whereas only acidic phosphate-limiting minimal medium induces *SPI-2* transcription [30]. Indeed, the *SPI-2* genes were neither induced nor essential under individual intramacrophage-mimicking stress conditions unless subjected to the interactive effects of diverse stress factors. Because of the complex environment inside macrophages, these intracellular niche-specific required genes reflected specialized essentiality under compound stress conditions.

After colonizing the small intestine, *S.* Typhimurium invades epithelial cells to gain access to the underlying lymph tissues. The invasion is mediated by a type III secretion system encoded at *SPI-1*. According to the Tn-seq results under HeLa selection, 23 of the 42 *SPI-1* genes were exclusively essential during epithelial invasion compared with other stresses in the intestine, including anaerobiosis, bile, low Fe^2+^, and osmolarity (Figure S3B). Although *S.* Typhimurium senses environmental signals in the intestine, such as anaerobic conditions, to induce transcription of *SPI-1* [30], the genes were only required during the invasion period, not to resist intestinal stresses.

### Identification of universal fitness factors for *S*. Typhimurium survival under different stress conditions

To identify genes that were essential under all the conditions, fitness determinants across all the output libraries were compared. We identified 12 genes (*dnaK*, *recB*, *recC*, *rnhA*, *pdxH*, *dcd*, *xseA*, *yfiO*, *yheM*, STM14_4641, *gidA*, and *orn*) with significantly decreased fitness levels under all the conditions studied (Table 1). Among the products encoded by these genes, the chaperone DnaK is a well-known ubiquitous stress-responsive protein to control cellular protein homeostasis under stressful conditions [31]. These stress-response proteins are essential under stress conditions and capable of triggering effective immune responses, making them potential immunogens for vaccine development [32]. For instance, DnaK proteins have been evaluated as target vaccine antigens for *Mycobacterium tuberculosis* because they are rapidly and abundantly expressed and induce strong T-cell regulation during *M. tuberculosis* infections [33].

**Table 1.** Genes exhibiting general fitness costs during all the stress conditions.

Gene ID	Gene	Product
STM14_0013	*dnaK*	molecular chaperone DnaK
STM14_0308	*rnhA*	ribonuclease H
STM14_1746	*pdxH*	pyridoxamine 5'-phosphate oxidase
STM14_2617	*dcd*	deoxycytidine triphosphate deaminase
STM14_3077	*xseA*	exodeoxyribonuclease VII large subunit
STM14_3264	*yfiO*	outer membrane protein assembly complex subunit YfiO
STM14_3609	*recB*	exonuclease V subunit beta
STM14_3611	*recC*	exonuclease V subunit gamma
STM14_4154	*yheM*	hypothetical protein
STM14_4641	–	putative reverse transcriptase
STM14_4671	*gidA*	tRNA uridine 5-carboxymethylaminomethyl modification enzyme GidA
STM14_5231	*orn*	oligoribonuclease

**Figure 6.**
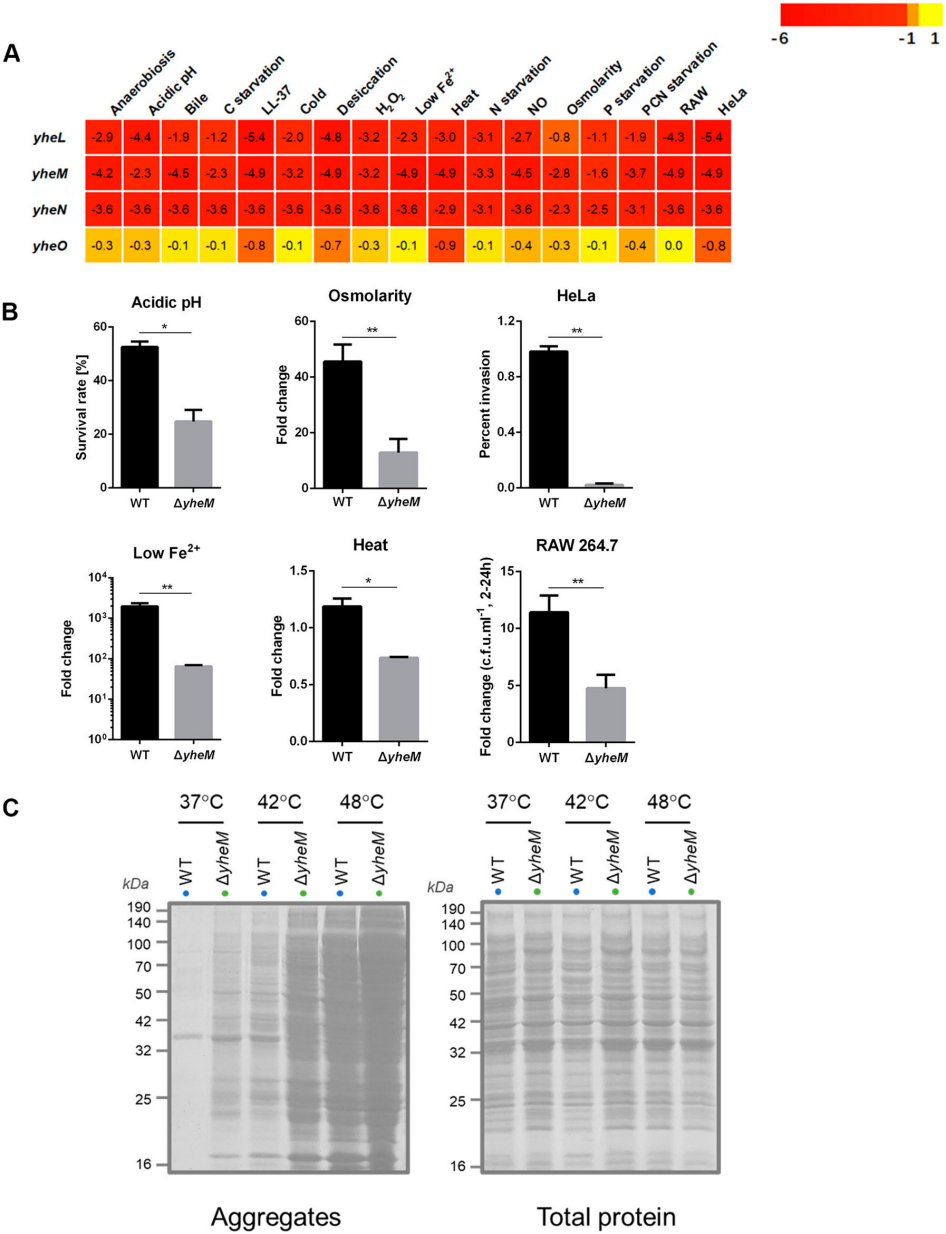
**Validation assays for survival and protein aggregation in *S*. Typhimurium lacking *yheM*.** (a) The average log_2_FC values across genes were shown for each condition. (b) Validation of the *in vitro* and cellular Tn-seq screening. Bacteria were treated under the same stress conditions as selecting the transposon mutant library. Viable cell counts were determined after stress for indicated time by plating serial dilutions. The data represent the mean values and standard errors from three independent experiments. *p values < 0.05, **p values < 0.01, Student’s *t*-test. (c) Protein aggregates isolated from wild-type and Δ*yheM* treated at 37°C, 42°C, or 48°C. Total cell lysates were used to indicate identical protein concentrations between samples.

Many genes listed in Table 1 encode well-defined enzymes or cellular component, while the function of *yheM* in *S*. Typhimurium remains unclear. In *E. coli*, *yheL*, *yheM*, and *yheN* (also named as *tusB*, *tusC*, and *tusD*) encode 3 sulfurtransferase subunits and form complex [34]. As a sulphur mediator, TusBCD is responsible for 2-thiolation of 5-methylaminomethyl-2-thiouridine (mnm^5^s^2^U_34_, 34 denotes the wobble position) in the wobble base of tRNA [34]. According to our Tn-seq results, the reads numbers of *yheLMN* were decreased significantly under all tested stresses (Figure 6(a)), while the adjacent *yheO* gene played no role in mnm^5^s^2^U modification [34] showed no difference in reads counts after stress treatments (Figure 6(a)). To confirm this Tn-seq result, we constructed *yheM* deletion mutant and determined the fitness under several stress conditions, including those of RAW 264.7 cells, HeLa cells, osmolarity, heat, acidic pH, and low Fe^2+^. In agreement with the Tn-Seq results, the *yheM* deletion mutant was more sensitive to these stresses compared to the wild-type strain (Figure 6(b)).

In *Saccharomyces cerevisiae* and *Caenorhabditis elegans*, cells lacking U_34_ 2-thiolation modifications exhibit slow codon translation rates, and slower decoding of discrete codons elicits widespread protein misfolding and accumulation of aggregated protein [35]. We hypothesized that *yheM* deletion mutant lost fitness in the stress conditions due to the defect in protein folding. We then treated the wild-type and *yheM* deletion mutant by heat shock (42°C and 48°C) to increase aggregated protein. In the wild-type background, aggregates were virtually undetectable at 37°C, and were strongly induced by heat shock (Figure 6(c)), which is consistent with earlier findings [21,36]. Strikingly, we found that deletion of *yheM* promoted the aggregation of endogenous protein at all three tested temperatures (Figure 6(c)). These data indicate that the loss of U_34_ modifications could result in aggregation of endogenous proteins, and further affect the fitness of bacteria under stress conditions.

In addition, we noticed that *recB* and *recC* were essential for *S.* Typhimurium under all the stresses tested. In *S*. Typhimurium, as in *E. coli* and other species, *recB*, *recC* and *recD* encode 3 exonuclease V subunits respectively and function as a complex that is involved in homologous DNA recombination [37]. Our Tn-seq results showed that the abundance levels of insertions in *recB* and *recC* were diminished notably in all the stress conditions (Figures S4A and S4B). However, the abundance level of insertions in *recD*, as well as the levels in the adjacent *ptr* genes, did not differ among the stress conditions. To validate the Tn-seq results, we constructed separate *recB*, *recC* and *recD* deletion mutants and determined their survival rates under several stress conditions, including those of macrophages, H_2_O_2_, bile, and NO. Consistent with the Tn-Seq results, both the *recB* and *recC* mutants were sensitive to these stresses, but the survival rate of *recD* mutant was comparable to that of the wild-type strain (Figure S4C).

Using the Tn-Seq approach, we defined more stress response-related genes that are potentially attractive targets for vaccine development.

### Identification of sRNAs essential for fitness

sRNAs contribute to post-transcriptional control of gene expression in *Salmonella* [38], and thus play important roles in carbon metabolism, virulence [39], and host–pathogen interactions [40]. Our previous study [41] identified 328 sRNAs in *S.* Typhimurium strain 14028s on the basis of studies conducted in *S*. Typhimurium strain SL1344 [40] and strain 4/74 [30]. To determine the fitness of these sRNAs when exposed to different stimuli, we analyzed the essentiality of these sRNAs under all the tested stress conditions.

**Figure 7.**
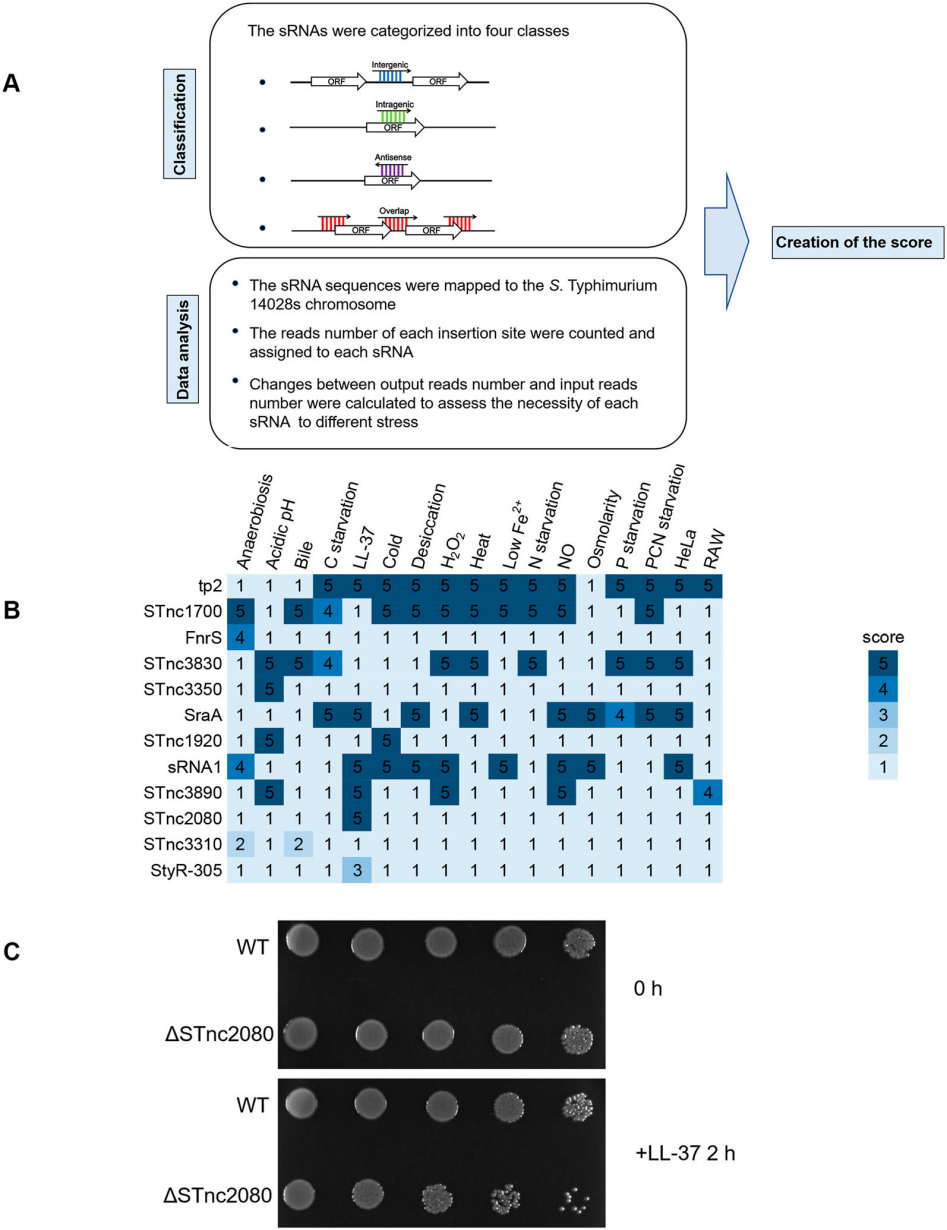
**Essentiality analyses and verification of sRNAs in *S*. Typhimurium.** (a) Flow-chart of the methods used for the sRNA analysis. (b) The sRNA scores under different stress conditions. (c) Overnight cultures of wild-type and ΔSTnc2080 cells in LB medium were diluted to OD_600_ of 0.1 in M9CA medium supplemented with 40 μg/ml LL-37. At the indicated time points, an aliquot of each culture was serially fivefold diluted and spotted onto LB plates. Plates were photographed after 14 h of incubation at 37°C.

The flowchart of the sRNA analysis strategy is summarized in Figure 7(a). First, 328 sRNAs were categorized into four groups (intergenic, antisense, overlapped, and intragenic) on the basis of their chromosomal locations [30]. For antisense, overlapped and intragenic sRNAs, a certain transposon insertion may affect both sRNAs and CDSs. To avoid this occurrence, the reads numbers of each insertion from the original Tn-seq data were assigned and counted only using the sequence database of 328 sRNAs instead of the CDS database. Then, Log_2_ FC values were calculated for each sRNA between the input and output samples as described previously (Dataset S3).

We established a scoring system to evaluate sRNA essentiality under each stress condition on the basis of log_2_FC values of reads as well as chromosomal location. The more significant the log_2_FC values of reads and the less interference by nearby coding genes resulted in higher scores. Scores were assessed on a 1−5 scale as follows: 1, log_2_FC in reads > −0.5 or *p* values > 0.05 regardless of sRNA position; 2, log_2_FC in reads ≤ −0.5 and *p* values < 0.05, antisense, intragenic or overlapped sRNA with interference by nearby coding genes; 3, log_2_FC in reads ≤ −0.5 and *p* values < 0.05, antisense, intragenic or overlapped sRNA without interference by nearby coding genes; 4, −1.0 ≤ log_2_FC in reads ≤ −0.5 and *p* values < 0.05, intergenic; 5, log_2_FC in reads < −1.0 and *p* values < 0.05, intergenic. Log_2_ FC and *p* values per sRNA were detailed in Dataset S3. For example, STnc3310 has a 125-bp overlap with the upstream gene *pflA*. After selection by anaerobic growth, the read abundances of six transposon insertion sites in STnc3310 were significantly decreased (Figure S5A). However, the read abundances of four sites in overlapped region, as well as the read abundances of insertion sites in other coding regions of *pflA*, also decreased. Because of the uncertain essentiality of STnc3310 under anaerobic stress condition, the score of STnc3310 was 2 (Figure 7(b)).

Another example is StyR-305, which is antisense within *ybhA* (Figure S5B). After the LL-37 treatment, the read abundances of two insertions located in StyR-305 significantly decreased (log_2_FC = −3.67, Dataset S3), whereas the read abundances of other insertions in the *ybhA-*coding region showed no significant changes. Although whether StyR-305 was essential under LL-37stress was still uncertain, StyR-305 underwent less interference by nearby coding genes as determined by analyzing read abundances coupled with the locational information. As a result, the score of StyR-305 under LL-37 stress condition was 3 (Figure 7(b)).

Using this scoring system, 10 sRNAs (tp2, STnc1700, FnrS, STnc3830, STnc3350, SraA, STnc1920, sRNA1, STnc3890, and STnc2080) with highest scores in at least one condition, were identified (Figure 7(b)). We chose STnc2080, which scored as high as 5 under LL-37 stress condition, to validate the Tn-seq results. STnc2080 deletion mutant strain was generated, and its viability under LL-37 stress condition was tested. As expected, the STnc2080 mutant strain was much more sensitive to LL-37 than the wild-type strain (Figure 7(c)).

## Discussion

### Advantages of screening and analysis strategies

In the two cellular selections used in this study, we made improvements to both screening and analysis, we conducted three rounds of continuous selection and calculated the weighted average FC of each gene (the weight of each round was 6:3:1). Thus, our results were more reliable compared with previous reports. A study using a microarray-based transposon tracking strategy suggested that both *SPI*-*1* and *SPI-2* were essential for *Salmonella* intracellular replication [42]. However, only the *SPI-2* locus is proved to be indispensable for intracellular *Salmonella* replication [29], which was confirmed in our results. The strategy of multiple selection and calculation of weighted average FC may explain the difference. The tapering weight distribution buffered the extreme read increase or loss in the third round but retained the variational tendency. Thus, compared with a one-round selection method, this three-round selection method allowed the identification of most fitness determinants during intracellular infection.

The gene *trxA* is another example. *TrxA* encodes thioredoxin, and a lack of thioredoxin produces a pronounced decrease in intracellular replication [43]. However, *trxA* was defined as “not required” in a previous Tn-seq-based work that selected the *S*. Typhimurium mutant library in macrophages by one-round selection [15]. Here, after three rounds of selection in RAW 264.7 cells, *trxA* was found to be essential. The known requirement for *trxA* in intramacrophage survival is consistent with the Tn-seq results and validates the three-round approach used in this model.

For genes that are not represented in the output pool, we judged their properties based on whether they have insertions in the input pool. Genes that have no insertions in either the output or the input sample are considered essential for growth because they do not tolerate insertions. Genes that have insertions in the input sample, but no or less insertions in the output sample are assumed to be important for fitness in the test conditions. In the current study, we did not focus on genes that are essential for growth. In *E*. *coli*, essential genes have been assayed by gain-of-function screens that use libraries of transposons with outward-facing promoters to facilitate gene overexpression [44]. In the future, we can also use this strategy to identify more essential genes in *Salmonella*.

### Levels of ATP demand varied in different niches

According to the comparative analysis, genes involved in ATP biosynthesis showed different essentiality in three mimicking host niches. In gastric niche, neither oxidative phosphorylation nor substrate-level phosphorylation was essential, which means even if the function of F_1_F_0_ ATP synthase was restrained, small amount of ATP generated by substrate-level phosphorylation was enough. A previous study showed that the intracellular ATP content of *S*. Typhimurium under acidic condition is equivalent to that under neutral culture conditions [45], suggesting that ATP does not play a key role in the adaptability of *S*. Typhimurium under the acidic condition in the stomach. Compared to gastric niche, ATP generated by oxidative phosphorylation is more important in intestinal niche. Even though the electron-transfer complexes were impaired, *S.* Typhimurium could still survival unless the irreplaceable F_1_F_0_ ATP synthase was abolished. Since the bacterial intracellular ATP concentration is crucial for *S.* Typhimurium proliferation in macrophages, every component responsible for ATP biosynthesis is essential.

The *atp* operon, encoding the subunits of F_1_F_0_ ATP synthase, is essential for the anaerobic proliferation of *Listeria monocytogenes* [46] and required in NO resistance of *Staphylococcus aureus* [47]. Our Tn-seq results were consistent with these reports: the *atp* operon was identified as essential under anaerobic and NO stress conditions. It is remarkable that the F_1_F_0_ ATP synthase is a bifunctional enzyme which not only synthesizes ATP but also exports protons coupled with ATP hydrolysis [48]. In *S*. *aureus*, F_1_F_0_ ATP synthase effectively extrudes protons and raises the intracellular pH above 8.0. The mildly alkaline cytosol is required for NO resistance [47]. As a result, it seems that both ATP hydrolysis and synthesis activities are responsible for the adaptation to stress conditions.

The *cydA* and *cydB* encode subunits of the cytochrome *bd* terminal oxidase [49] and act as an electron-transfer complex in oxidative phosphorylation. The complex has high NO dissociation rate and notable catalase activity and is required for *Salmonella* to defend against nitrosative or peroxidative stress [50]. Our results are in line with these reports that *cydA* and *cydB* are essential in NO and H_2_O_2_ stresses, and explain the essentiality of cytochrome *bd* complex in an intracellular niche. However, the specific mechanism in which the *cydA* and *cydB* are also essential in gastric and intestinal stresses remains to be determined.

### Novel role of tRNA modifications in bacterial stress response

5-methylene-2-thiouridine derivative modifications in the wobble position of tRNAs is conserved both in bacteria and Eukarya [51]. In *E*. *coli*, several enzymes are responsible for the biosynthesis of the 2-thiolation modification of mnm^5^s^2^U_34_ besides TucBCD complex (named YheLMN in *Salmonella*), including IscS, MnmA [52], TusA, and TusE [34] (named NifS, TrmU, YhhP, and YccK in *Salmonella*, respectively). According to our Tn-seq results, *yheLMN* showed general essentiality under nearly all the stresses. Moreover, *nifS*, *trmU*, and *yhhP* were required under at least 12 of the 17 stress conditions, indicating 2-thiolation modification of mnm^5^s^2^U_34_ is indispensable for *Salmonella* to respond to environmental stresses.

GidA and TrmE are responsible for the addition of the (c)mnm^5^ group onto U_34_ of tRNAs [53]. Consistent with genes involved in 2-thiolation modification onto U_34_, both *gidA* and *trmE* were required under most of the stress conditions (Table 1 and Dataset S2). This result further highlights the role of modification in the wobble position of tRNA in bacterial stress response.

Lack of *dnaK* also results in a strong increase in the aggregation of protein [21]. Protein aggregation can result in a loss of protein function, protein homeostasis perturbation, and interference with a wide array of cellular processes [54]. Proteins that have been identified in aggregates include those associated with carbon metabolism, oxidative phosphorylation, translation, DNA replication, and repair [21,55]. To prevent and resolve protein aggregation, bacteria have evolved protein quality control systems composed of molecular chaperones and proteolytic machineries [56]. Moreover, bacterial ClpB-DnaKJE bichaperone system is the primary disaggregation machinery to revert protein aggregation in the cytosol of bacteria [56]. However, acute stresses and loss of certain cellular function can overwhelm the proteostasis network. In this study, we showed *Salmonella* lacking *yheM* accumulated more aggregated proteins under stress conditions. We postulate that tRNA modification defects may contribute to the disruption of protein homeostasis network utilized by *Salmonella* in multiple stress conditions.

### RecBC is a universal fitness determinant

Both Tn-seq results and mutant phenotypic assay showed that RecB and RecC were required under all stress conditions, while RecD was not. When RecD is lacking, productive recombination and DNA repair still occur but the nuclease activity of the enzyme significantly decreases [57]. Furthermore, the role of *recD* in recombination and repair appears to be redundant because recombination in strains lacking *recD* is heavily dependent on the activities of other exonucleases, such as RecJ and exonuclease VII [58]. The gene encoding the exonuclease VII large subunit, *xseA*, was required under all the stress conditions (Table 1), whereas *recJ* was dispensable under different stresses (Dataset S2). However, *Salmonella* lacking both *recD* and *recJ* are attenuated in mice and unable to proliferate in macrophages [59]. Therefore, we speculate that the *recD* mutant strain showed resistance to different kinds of stresses because the deficiency in holoenzyme activities was compensated for by other exonucleases.

### Developing new strategy to investigate sRNAs by Tn-Seq.

A single transposon insertion may result in the inactivation of both the sRNA and coding gene. As a result, there are limited investigations of sRNAs using Tn-Seq. Here, we established a strategy by synergistically analyzing the fitness of sRNAs under various conditions and their genomic locations. This strategy avoided potential conflicts when a certain read was assigned to both a CDS and sRNA and were able to evaluate the essentiality of sRNAs under different conditions simultaneously.

A total of 10 sRNAs were identified as essential under at least one condition. Among them, tp2 is required for *Salmonella* growth in both nutritious and minimal media [15], which is in accordance with its high score of 5 under the four starvation conditions using our sRNA analysis system (Figure 7(b)). In addition to tp2, FnrS is an anaerobically induced sRNA in *E. coli* that adjusts gene expression for anaerobic growth [60], which is in line with its score of 4 under anaerobic stress (Figure 7(b)). These two cases strongly supported the effectiveness of our screening and scoring system. The STnc2080 mutant strain was more sensitive to LL-37 stress, which again confirmed the effectivity of our scoring system. Therefore, this strategy provided a way to investigate the essentiality of sRNAs using Tn-Seq.

### Perspective

The capacity of *S.* Typhimurium to thrive under a variety of stress conditions is related to virulence. To fully understand how *S.* Typhimurium manipulates virulence factors in response to certain environmental stimuli both *in vivo* and *in vitro*, it is important to determine *S.* Typhimurium gene dependency under different stress conditions. This report represents a comprehensive resource that demonstrates fitness determinants or sRNAs in 2 cell lines and under 15 *in vitro* conditions. More importantly, a novel essential fitness protein named YheM was identified and could be potential for vaccine development to control *Salmonella* infection. Investigating adaptations of *S*. Typhimurium to *in vitro* stresses increases our understanding of the strategies that bacteria exploit to colonize and proliferate *in vivo*. However, the conditions and cell lines we chose cannot fully mimic *in vivo* environments. In the future, it will be important to further explore the genetic requirements of *S*. Typhimurium under *in vivo* conditions and, especially, to investigate the genetic requirements of *S*. Typhimurium in different host organs to understand organ-specific niches and nutritional requirements.

## Supplementary Material

Supplemental Material
