# Supplementary material for: Gene essentiality profiling reveals a novel determinant of stresses preventing protein aggregation in Salmonella
Source: Emerg Microbes Infect. 2022 Jun 4;11(1):1554–71. doi: 10.1080/22221751.2022.2081618 (PMC9176671; doi:10.1080/22221751.2022.2081618)
Supplement: Supplemental Material [file TEMI_A_2081618_SM5510.zip › Supporting information.docx]

Supporting Information

**
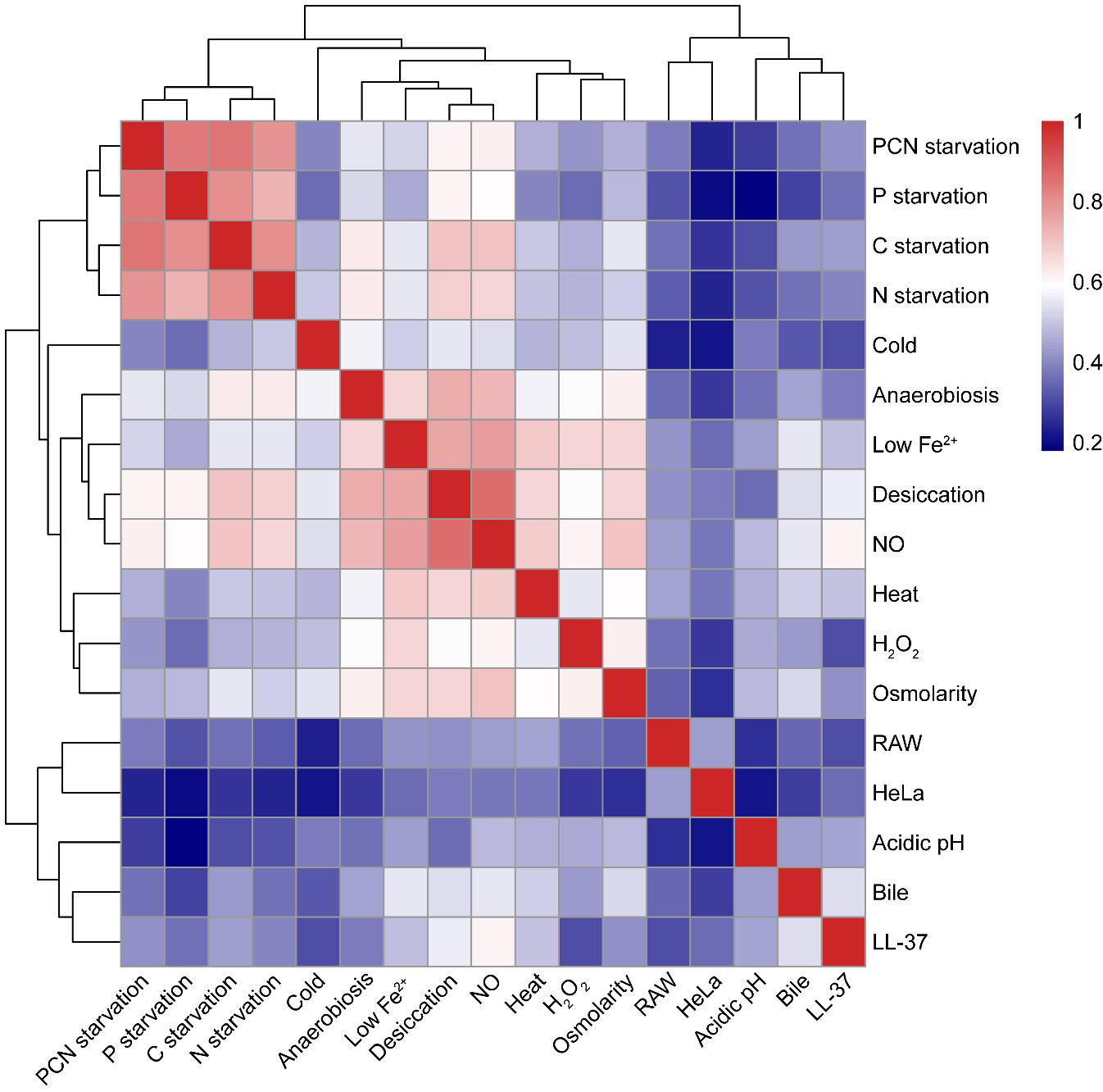
**

**Figure S1.** **17 conditions cluster according to similarity of fitness contribution per gene.** Samples were hierarchically clustered and their Pearson’s correlation coefficients were illustrated using a heatmap. Sample-to-sample Pearson’s correlation coefficients were calculated on the basis of their log_2_ FC distributions.





**Figure S2. Validation of Tn-Seq results.** The replication folds in RAW 264.7 cells were determined to evaluate the bacterial intracellular fitness. The net growth between 2 h and 24 h was calculated from the fold change in CFUs at these time points. **p* values < 0.05, ***p* values < 0.01, Student’s *t*-test.

A

**
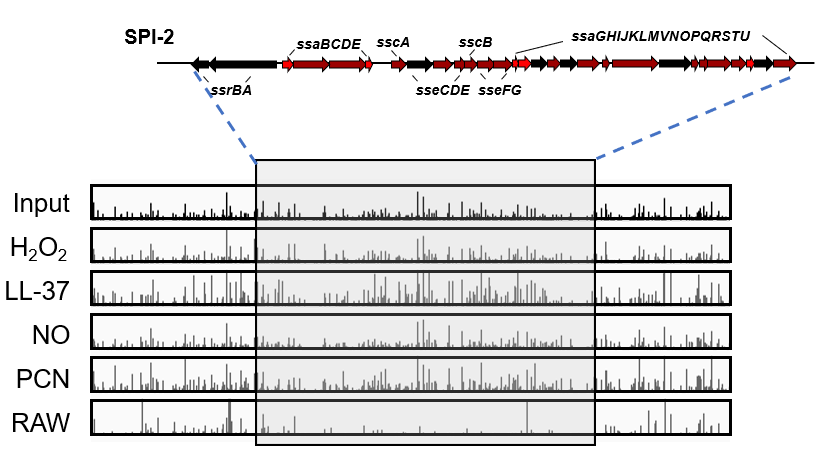
**

B


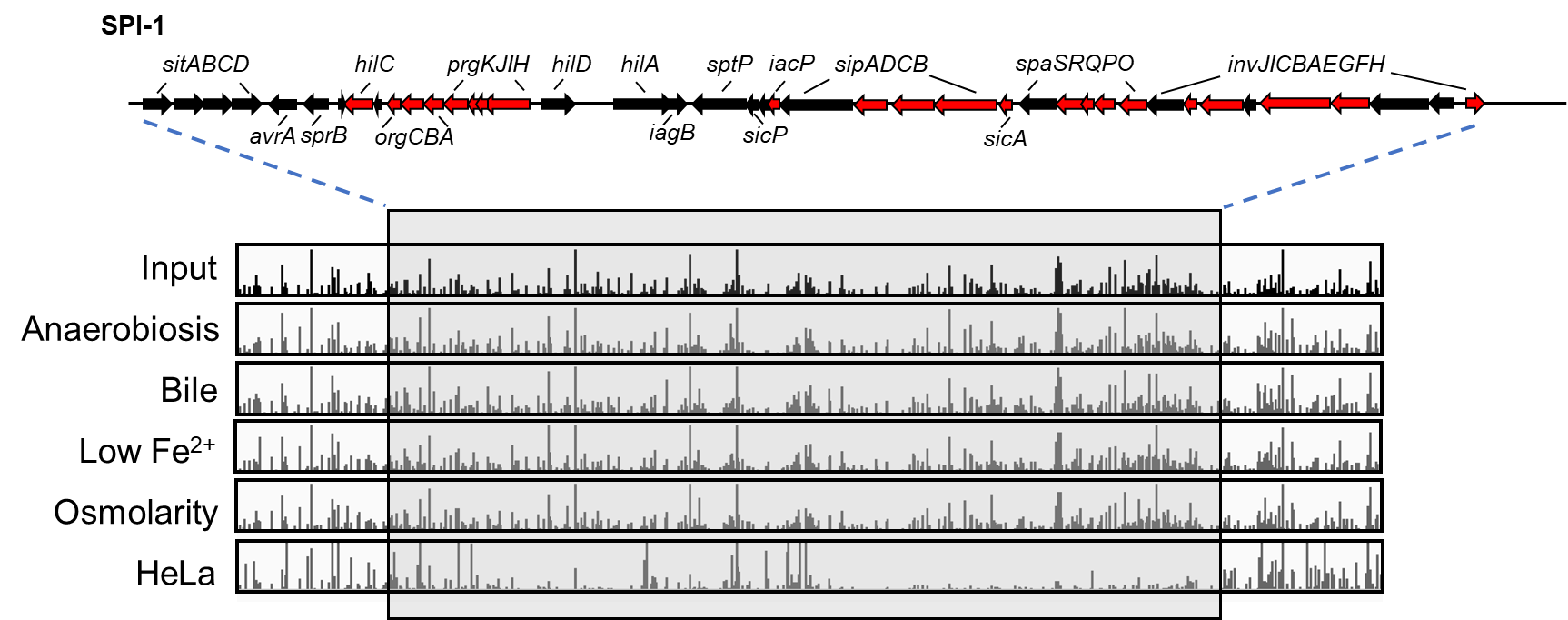


**Figure S3.** **Visualization of mapped sequence reads in the SPIs.** (A) Mapped sequence reads for the *SPI-2* genes presented in the IGV browser. The heights of the bars correspond to the numbers of reads. Fitness determinants for survival in macrophages were labeled in red; genes exclusively essential in macrophages were labeled in dark red. (B) Mapped sequence reads for the *SPI-1* genes presented in the IGV browser. The heights of the bars correspond to the numbers of reads. Genes essential for invading HeLa cells were labeled in red.


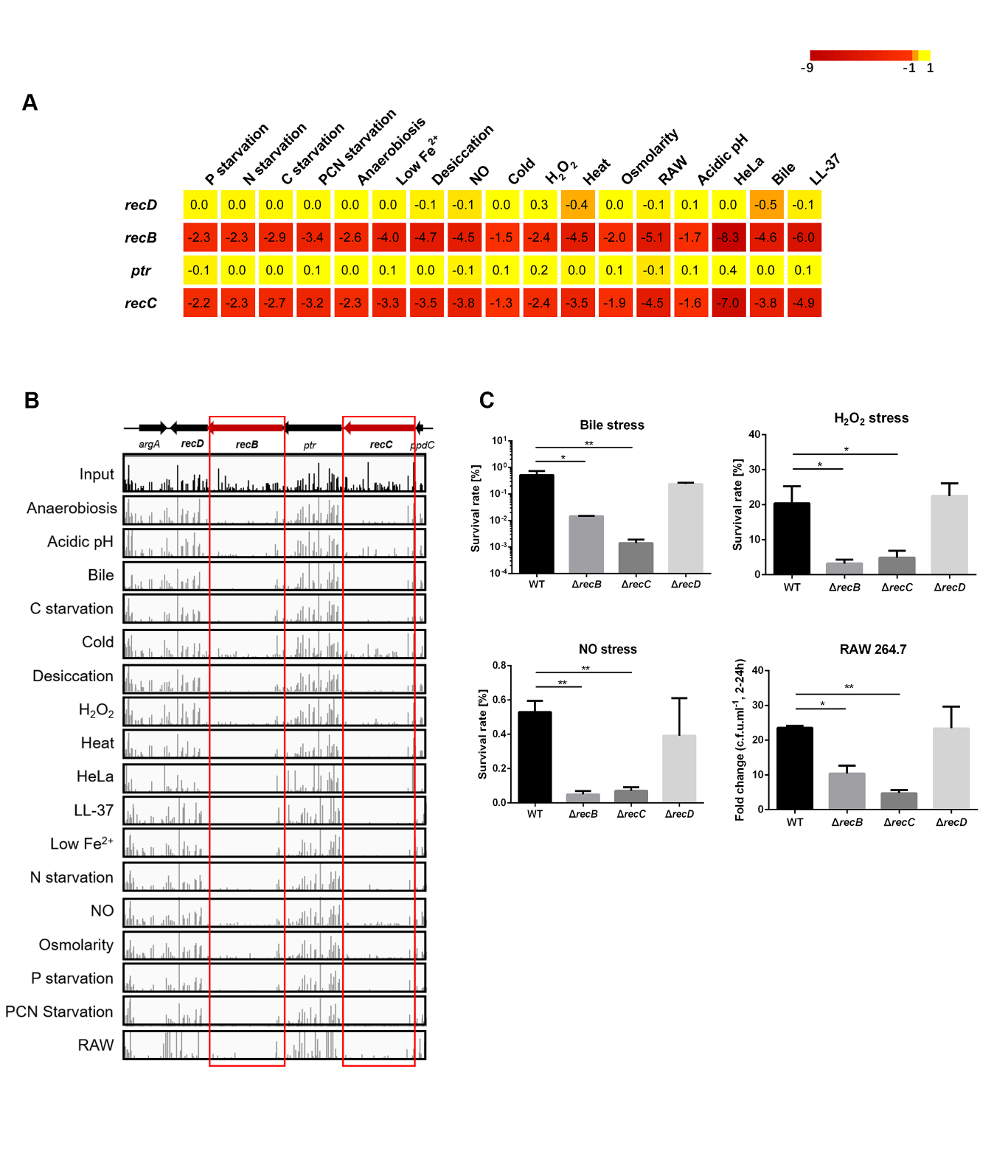


**Figure S4. Identification of RecBC as a general stress factor.** (A) The average log_2_FC values across genes were shown for each condition. (B) Mapped sequence reads for *recB*, *recC*, *recD* and adjacent genes presented in the Integrative Genomics Viewer (IGV) browser. The heights of the bars correspond to the numbers of reads. (C) Validation of the *in vitro* and cellular Tn-seq screening. The survival rates of the *recB*, *recC* and *recD* knockout strains were determined under selected stresses (Table 1). The data represent the mean values and standard errors from three independent experiments. **p* values < 0.05, ***p* values < 0.01, Student’s *t*-test.

A


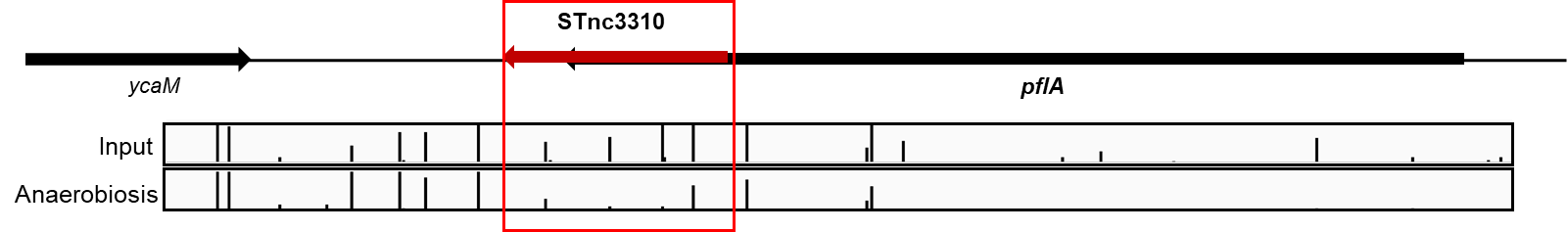


B


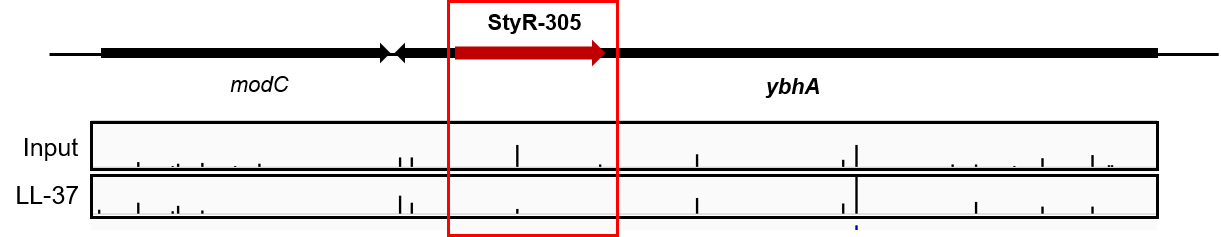


**Figure S5.** **Visualization of Mapped sequence reads in sRNAs.** (A) Mapped sequence reads for the overlapped sRNA STnc3310 and nearby genes presented in the IGV browser. (B) Mapped sequence reads for the antisense sRNA StyR-305 and nearby genes presented in the IGV browser. Protein coding gene were labeled in black, sRNA was labeled in red. The heights of the bars correspond to the numbers of reads.

**S1 Dataset**. **Statistics for Tn-seq analysis.** The file gives the number of sequenced reads at every step and the number of insertions in CDS and intergenic regions for each sample.

**S2 Dataset**. **Analysis of coding genes for all conditions.** The file gives transposon insertion sites, number of reads, log_2_ FC and *p* values per gene in the input sample and output samples for each condition. Fitness determinants under each condition were remarked as “essential”.

**S3 Dataset**. **Analysis of sRNAs for all conditions.** The file gives transposon insertion sites, number of reads, log_2_ FC and *p* values per sRNA in the input sample and output samples for each condition.
